# Dosimetric advantages of non-coplanar volumetric modulated arc therapy (VMAT) using biaxially rotational dynamic radiation therapy compared with coplanar VMAT in prostate stereotactic body radiation therapy with focal boost

**DOI:** 10.1007/s11604-026-01974-y

**Published:** 2026-04-30

**Authors:** Rihito Aizawa, Yuka Ono, Hideaki Hirashima, Takashi Ogata, Shinya Hiraoka, Mitsuhiro Nakamura, Takashi Mizowaki

**Affiliations:** 1 Department of Radiation Oncology and Image-Applied Therapy, Graduate School of Medicine, Kyoto University, 54 Kawahara-cho, Shogoin, Sakyo-ku, Kyoto 606-8507 Japan; 2 Department of Advanced Medical Physics, Graduate School of Medicine, Kyoto University, 53 Kawahara-cho, Shogoin, Sakyo-ku, Kyoto 606-8507 Japan

**Keywords:** Prostate cancer, Biaxially rotational dynamic radiation therapy, Stereotactic body radiation therapy, Focal boost, Non-coplanar trajectory

## Abstract

**Purpose:**

To identify dosimetric advantages of biaxially rotational dynamic radiation therapy (BROAD-RT) for stereotactic body radiation therapy (SBRT) with focal boost for non-metastatic prostate cancer (PCa), compared with coplanar volumetric-modulated arc therapy (co-VMAT).

**Materials and methods:**

BROAD-RT is a unique beam delivery technique, which facilitates sequential non-coplanar beam delivery without the need to rotate the couch or reposition the patient. For 15 patients with non-metastatic PCa, two different plans (BROAD-RT and co-VMAT) were created, and these plans and dosimetric indices were compared. The prescribed dose was 35 Gy in 5 fractions to the whole prostate gland, and that to the intra-prostatic dominant lesions (IPDLs) was increased up to 50 Gy. Quality assurance (QA) was performed for BROAD-RT and co-VMAT plans from the 15 patients, and the calculated and measured dose distributions were evaluated according to global gamma analysis using ArcCHECK.

**Results:**

The dose coverage of the target volumes and the high-dose exposure to organs at risk (rectum, bladder, and urethra) were not significantly different between BROAD-RT and co-VMAT plans. The normal tissue dose outside of the planning target volume (PTV) (maximum dose 2 cm away from PTV [D2 cm], and 30%, 50%, or 70% isodose volume divided by the volume of PTV [R30, R50, and R70]) were significantly lower in the BROAD-RT plan (*p* < 0.001, *p* < 0.001, *p* < 0.001, and *p* < 0.001, respectively). In QA, average (± standard deviation) passing rates were 98.2 ± 0.7% for BROAD-RT and 99.0 ± 1.0% for co-VMAT.

**Conclusion:**

Non-coplanar VMAT via BROAD-RT improved the dose distribution, mainly outside of the PTV and for some of the organs at risks, in prostate SBRT with focal boost compared with coplanar VMAT. Therefore, as BROAD-RT enables practical implementation of non-coplanar VMAT, it is considered a promising radiotherapy method of SBRT with focal boost for non-metastatic PCa.

**Supplementary Information:**

The online version contains supplementary material available at 10.1007/s11604-026-01974-y.

## Introduction

Prostate cancer (PCa) is the second most common cancer in males [1], and the incidence rate has shown a continual increase [2]. Intensity-modulated radiation therapy (IMRT) is one of the standard treatment modalities for non-metastatic prostate cancer (PCa) [3–5]. Although IMRT can help achieve excellent disease control [6–10], local intra-prostatic recurrence is an important recurrence pattern that needs to be resolved following IMRT for PCa, which has been reported to primarily occur at the site of the primary tumor (intra-prostatic dominant lesions [IPDLs]) [11, 12]. The effectiveness of selective focal dose escalation for IPDLs, using the simultaneous-integrated boost (SIB) method, has been reported as one of the promising IMRT methods for safe dose-escalation [13–18]. This IMRT method has been also applied to stereotactic body radiation therapy (SBRT) [13]. Owing to its radiobiologic benefits based on the reportedly low alpha-beta ratio of PCa [19] and benefits regarding both patients’ convenience and medical economics, hypofractionated EBRT has been widely applied in recent clinical practice [20], and a representative example is the hypo-FLAME trial. The hypo-FLAME phase 2 trial demonstrated the safety of SBRT with focal dose-escalation to IPDLs, in which 35 Gy in 5 once-weekly fractions was prescribed to the entire prostate with an integrated boost up to 50 Gy to IPDLs [21].

SBRT usually uses beams from non-coplanar aspects to improve dose conformity to targets. Indeed, the use of non-coplanar beams has proven advantageous in organs at risk (OARs) dosimetry for several tumor types [22–27], consequently having the potential to reduce RT-induced toxicities. In prostate SBRT, non-coplanar beam uses significantly reduced the dose to OARs, such as the rectum or bladder, in volumetric-modulated arc therapy (VMAT) for PCa [28, 29]. However, the application of non-coplanar beams using a commonly available C-arm linear accelerator requires the rotation of the couch and reposition the patient, rendering it difficult its application in prostate IMRT using a commonly available C-arm linear accelerator in clinical practice.

**Fig. 1:**
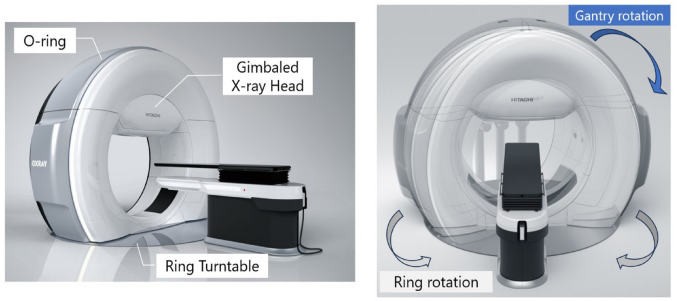
View of the OXRAY system. One distinctive feature is the ability to move both rotation axes (gantry and ring) simultaneously, which facilitates continuous beam delivery of non-coplanar VMAT without the need to rotate the couch or reposition the patient. VMAT, volumetric modulated arc therapy

Recently, the 2nd generation O-ring shaped linear accelerator, OXRAY (Hitachi High-Tech Corporation), was launched (Fig. 1), which facilitates a unique beam delivery technique, biaxially rotational dynamic radiation therapy (BROAD-RT) (Dynamic SwingArc^®^) [30–33]. This RT technique enables sequential non-coplanar beam delivery without the need to rotate the couch or reposition the patient, and therefore enables an easy application of non-coplanar beams in clinical practice. We previously performed a prospective pilot study to evaluate the feasibility of hypofractionated IMRT with focal boost using BROAD-RT for patients with high-risk PCa, and reported its usefulness and safety [16]. Herein, we performed a dosimetric planning study to investigate dosimetric advantages of BROAD-RT over coplanar VMAT (co-VMAT) in the setting of SBRT, which requires higher dose conformity.

## Materials and methods

This study followed the tenets of the Helsinki Declaration, with approval from the institutional ethical review board (approval number: R1048-3). Written informed consent was not required due to the nature of the study, solely using CT and MRI data.

### Overview of OXRAY system

We previously reported details of the OXRAY system [30, 31]. In brief, it is a novel linac system with an O-ring structure, and one of its distinctive features is the ability to move both rotation axes (gantry and ring) simultaneously, which facilitates continuous beam delivery of non-coplanar VMAT without the need to rotate the couch or reposition the patient (Fig. 1).

### Patient selection and contouring

Planning CT images (2 mm thick) from 15 clinically treated patients who received IMRT with focal boost to IPDLs for non-metastatic PCa between February 2023 and August 2023 were used for the current study. These 15 patients were retrospectively and randomly selected from the 29 patients who received IMRT with focal boost to the IPDLs during this period.

The targets (prostate, seminal vesicles, and gross tumor volume [GTV]) and OARs (rectum, bladder, urethra, and bowels) were the same as in the clinical plans. The margins for the targets and OARs and dose constraints were the same as those in the hypo-FLAME phase 2 study [21]. IPDLs visible on pre-treatment MRI were contoured as GTV in consideration of the PI-RADS ver.2 recommendation [34]. The clinical target volume (CTV) consisted of the whole prostate gland plus 4 mm around GTV excluding OARs (CTV_prostate), and the base (intermediate-risk) or proximal two-thirds (high-risk) of the seminal vesicles (CTV_SV). The 4 and 5-mm margins for the planning target volume (PTV) were added around CTV_prostate and CTV_seminal vesicles isotopically, creating PTV_prostate and PTV_seminal vesicles, respectively. PTV_all was defined as PTV_prostate plus PTV_seminal vesicles. As OARs, the rectum, bladder, urethra, femoral heads, penile bulb, and small/large bowel (if attached to PTV) were delineated based on CT. A 2-mm margin for the planning organ at risk volume (PRV) was added around the rectum and urethra isotopically to create PRV_rectum and PRV_urethra, respectively. In addition, we created PTV-GTV5, which was the volume after subtracting the volume expanded 5 mm around the GTV from PTV_prostate, to evaluate conformity of focal boosting (Table 1).

**Table 1:** Patient characteristics

Characteristics		
Number of patients	15	
Age at IMRT [y], median (range)	74	(68–85)
Initial PSA [ng/mL], median (range)	13.85	(5.11–32.9)
Clinical T stage, n [%]		
T2a	6	40.0%
T2b	2	13.3%
T2c	3	20.0%
T3a	4	26.7%
Gleason score		
6	2	13.3%
7	6	40.0%
8	2	13.3%
9	5	33.3%

IMRT, Intensity-modulated radiation therapy; PSA, Prostate-specific antigen

Details of targets and OARs are shown in Supplementary Table 1.

### Treatment planning: dose constraints

Dose constraints of targets and OARs were the same as those in the hypo-FLAME phase 2 study [21]. DX% and DX cc were defined as the dose to X% and X cc of the target or OAR volume, respectively, and VX Gy was defined as the volume receiving at least X Gy. The prescribed dose was 35 Gy in 5 fractions to the whole prostate gland. The dose to PTV_prostate was 33.25 Gy (95% of 35 Gy). That to GTV was escalated up to 50 Gy (D0.1 cc ≤ 52 Gy) as long as the dose constraints regarding OARs were maintained. Details of dose constraints of targets and OARs are shown in Supplementary Table 1.

**Fig. 2:**
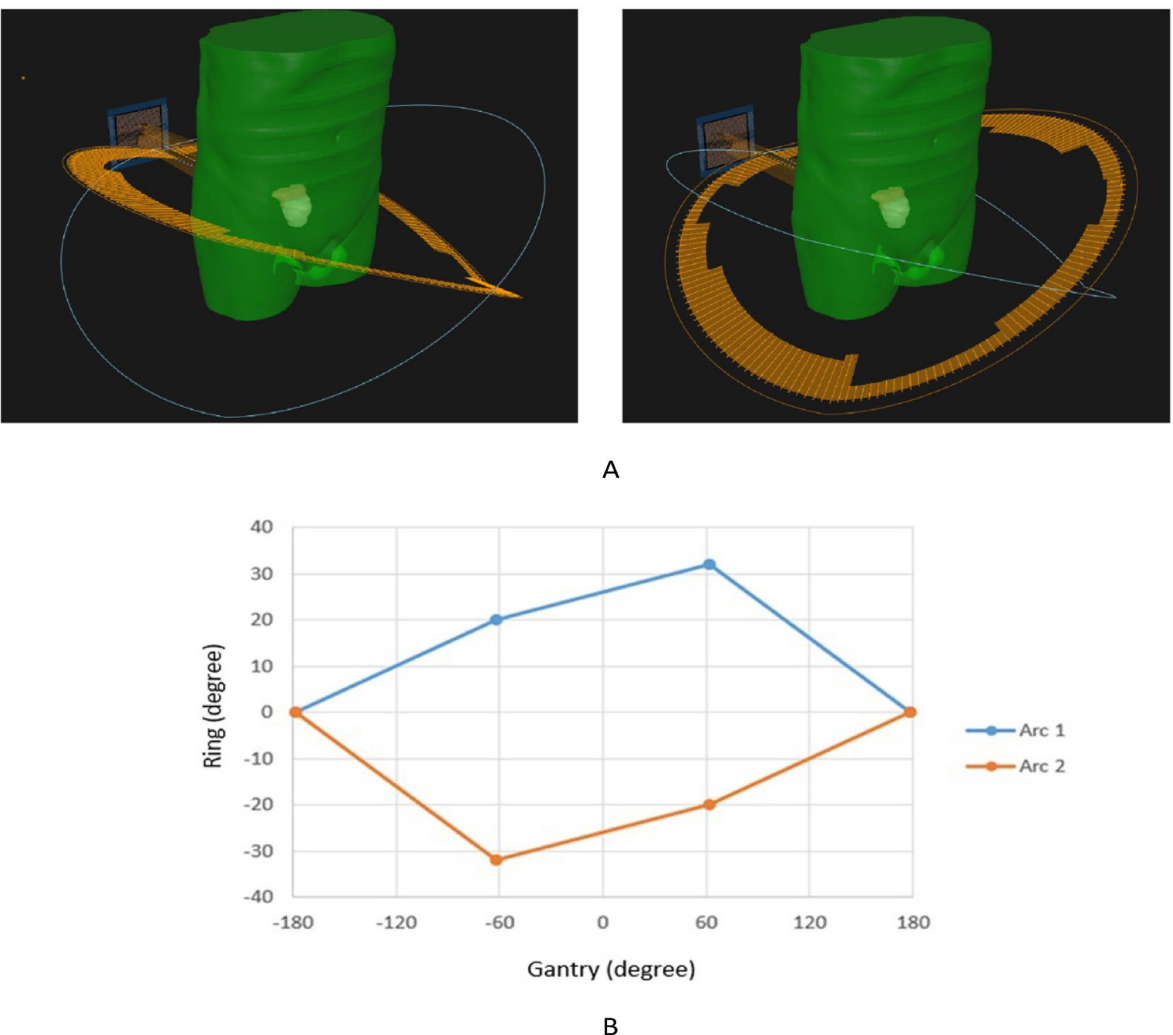
Arc trajectories of BROAD-RT (**A**). The diagrams show the relation between the gantry and O-ring rotational positions/degrees (**B**). BROAD-RT, biaxially rotational dynamic radiation therapy

BROAD-RT and co-VMAT treatment plans were created using RayStation version 2024B (RaySearch Medical Laboratories AB, Stockholm, Sweden). The BROAD-RT plan was created using two non-coplanar arc trajectories to form a “waving X shape,” intended to maximize the non-coplanar component while avoiding the collision between gantry and couch. More specifically, as the rotation angle of the gantry is limited due to the risk the patient’s body (or the couch) colliding with the gantry, we, therefore, selected the maximum ring rotation angle at four gantry points (−180, −60, 60, 180 degree), and connected them to create the trajectory to form the first arc. We then created a symmetrical mirror trajectory to form the second arc. The trajectories of BROAD-RT are presented in Fig. 2A, and the relation between the gantry and O-ring rotational positions/degrees are presented in Fig. 2B. The co-VMAT plan was created using two full coplanar arcs.

To minimize the bias of effort for plan creation, optimizations of both IMRT plans were performed using the same template, and the optimization process was repeated until the treatment plans met the dose constraints. In both plans, 6-MV photon beams and the collapsed cone dose calculation algorithm (version 5.1) was used for calculation with a grid size of 2.0 mm.

**Fig. 3:**
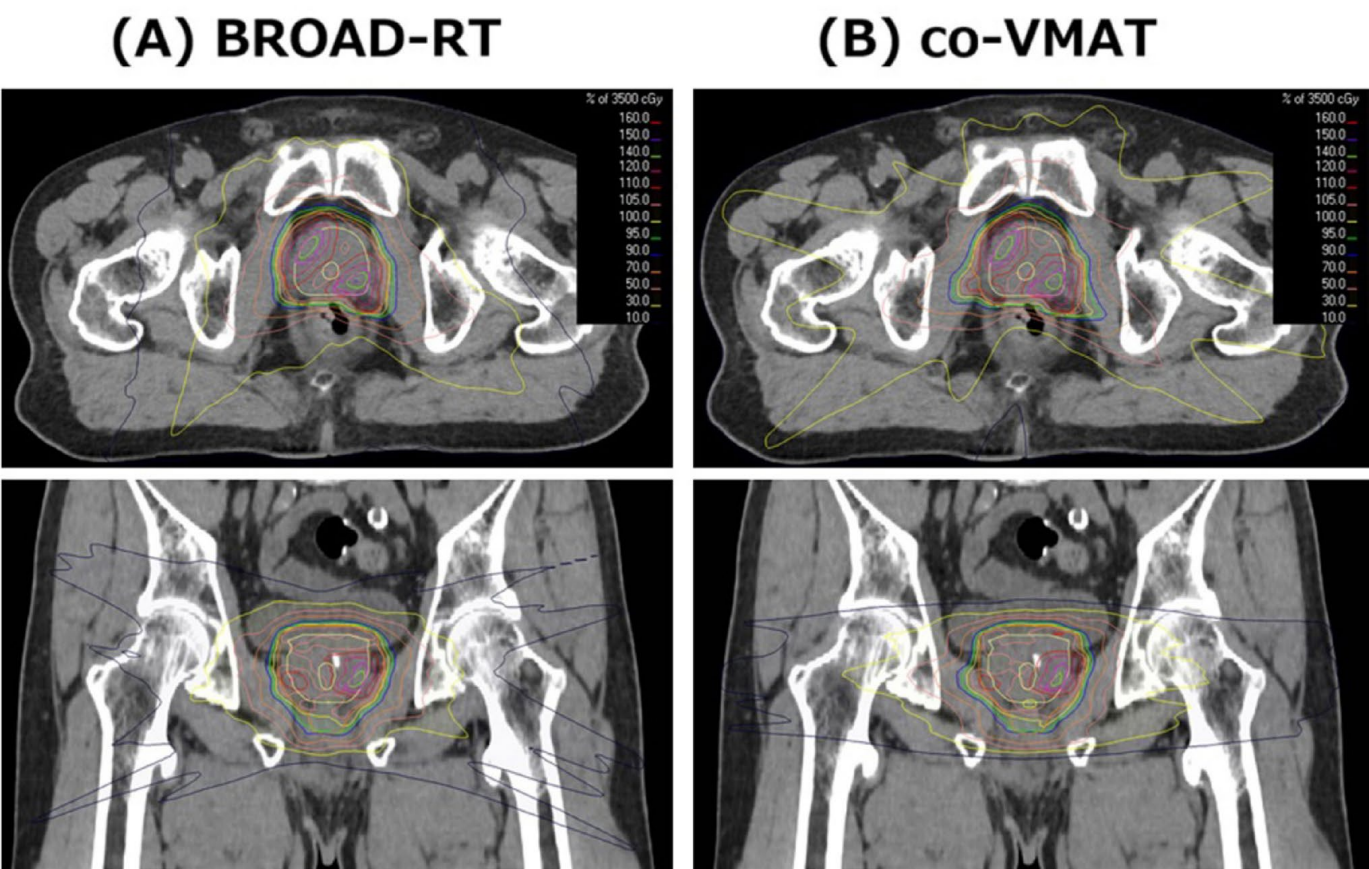
Dose distribution in representative cases of BROAD-RT (A) and co-VMAT (B) plans. Each colored line shows the targets. Key: yellow, clinical target volume of prostate (CTV_prostate); orange, planning target volume of prostate (PTV_prostate); purple, gross tumor volume (GTV); yellow, urethra. BROAD-RT, biaxially rotational dynamic radiation therapy; co-VMAT, coplanar volumetric modulated arc therapy; CTV, clinical target volume; PTV, planning target volume; GTV, gross tumor volume

Representative examples of the structure delineation and dose distribution are shown in Fig. 3.

### Plan evaluation and statistical analysis

Plan and dosimetric indices from BROAD-RT and co-VMAT plans were evaluated. The dosimetric indices used in the comparison were basically selected from dose constraints used in the hypo-FLAME phase 2 study [21]. In addition, the conformity index (CI) for GTV and PTV_prostate (target volume covered by 95% isodose [47.5 Gy for GTV and 33.25 Gy for PTV_prostate] divided by total 95% isodose volume), D0.03 cc of PTV-GTV5 (near maximum dose away from GTV plus 5 mm inside PTV) was evaluated, and D2cm (maximum dose 2 cm away from PTV_all), as well as R10, R30, R50 and R70 (10%, 30%, 50%, or 70% isodose line volume divided by volume of PTV_all, respectively) were evaluated to assess the dose gradient.

These dosimetric indices and number of optimizations of BROAD-RT and co-VMAT plans were compared using the Wilcoxon signed-rank test, and a p-value < 0.05 was regarded as significant. All statistical analyses were carried out using EZR version 1.61 [35], which is a graphical user interface for R version 4.1.2 (R Foundation for Statistical Computing, Vienna, Austria).

### Dosimetric quality assurance, total monitor units, and beam delivery time

Quality assurance was performed for BROAD-RT and co-VMAT plans from the 15 patients. The calculated and measured dose distributions were evaluated according to global gamma analysis using ArcCHECK (Sun Nuclear Corp., Melbourne, FL, USA), in which passing rates (3%/2 mm) with 10% dose threshold criteria were used [36]. In addition, total monitor unit (MU) and delivery time (measured from the beginning to end of irradiation including the ring operating time) were compared using the Wilcoxon signed-rank test.

## Results

### Target volume coverage, OAR doses, and optimization

Regarding dose coverage of target volumes, no significant differences were observed between BROAD-RT and co-VMAT plans. D0.03 cc of PTV-GTV5 was significantly lower in the BROAD-RT plan. Specifically, means of D0.03 cc were: PTV-GTV5, 42.09 Gy for BROAD-RT plan; 42.83 Gy for co-VMAT plan (*p* = 0.0067). Regarding high-dose exposure to OARs, no significant differences were observed between BROAD-RT and co-VMAT plans. Regarding medium dose exposure to OARs, such as D15% and D20% of the rectum, and D90% of the penile bulb were significantly lower in the co-VMAT plan (*p* < 0.001, *p* < 0.001, and 0.018, respectively), while D5% and the mean dose of the femoral head were significantly lower in the BROAD-RT plan (*p* < 0.001 and *p* < 0.001, respectively). Regarding normal tissue doses outside of PTVs, D2cm, R30, R50, and R70 were significantly lower in the BROAD-RT plan (*p* < 0.001, *p* < 0.001, *p* < 0.001, and *p* < 0.001, respectively), whereas R10 were significantly lower in the co-VMAT plan (*p* < 0.001). No significant difference in CI for GTV and PTV_prostate was observed. Table 2 shows the details of indices for targets and OARs.

The median value of optimization times was 11 for both BROAD-RT (interquartile range [IQR: 8.5–13]) and co-VMAT (IQR: 7–12.5) plans, with no significant difference noted (*p* = 0.23).

**Table 2:** Comparison of dosimetric parameters between BROAD-RT and co-VMAT plans

Structure	BROAD-RT	BROAD-RT co-VMAT	
	Mean ± SD	Mean ± SD	*p*-value
*GTV*			
D99% [Gy]	42.03±2.71	42.09±2.68	0.8
D0.1 cc [Gy]	51.17±0.68	50.98±0.79	0.52
CI	0.83±0.09	0.84±0.08	0.36
*CTV_prostate*			
D99% [Gy]	35.74±0.36	35.59±0.27	0.38
*PTV_prostate*			
D99% [Gy]	33.71±0.23	33.68±0.24	0.68
CI	0.68±0.06	0.69±0.065	0.077
*CTV_seminal vesivles*			
D99% [Gy]	34.52±0.93	34.81±0.72	0.19
*PTV_seminal vesivles*			
D99% [Gy]	31.30±0.60	31.25±0.62	0.56
*Rectum*			
D0.03 cc [Gy]	38.67±0.46	38.77±0.58	0.14
D1cc [Gy]	36.55±0.51	36.72±0.43	0.68
D2cc [Gy]	34.51±0.63	34.53±0.45	0.76
D15% [Gy]	22.1±3.34	20.95±3.28	<0.001
D20% [Gy]	17.93±2.83	16.64±2.75	<0.001
*PRV_Rectum*			
D0.03 cc [Gy]	40.72±1.21	40.75±1.20	0.76
*Urethra*			
D0.03 cc [Gy]	39.03±0.71	39.08±0.57	0.8
*PRV_Urethra*			
D0.03 cc [Gy]	41.34±0.45	41.19±0.59	0.25
*Bladder*			
D1cc [Gy]	37.46±0.48	37.58±0.59	0.14
D5cc [Gy]	36.28±0.66	36.20±0.60	0.17
D15% [Gy]	23.12±5.87	23.49±6.04	0.083
D20% [Gy]	19.11±5.48	19.42±6.35	0.095
*Penile bulb*			
D90% [Gy]	7.03±2.89	5.94±3.65	0.018
*Femoral head*			
D5% [Gy]	9.33±1.89	14.43±1.66	<0.001
Mean	5.67±1.27	8.64±1.75	<0.001
*PTV-GTV5*			
D0.03 cc [Gy]	42.09±0.89	42.83±1.03	0.0067
D2cm [Gy]	23.14±1.27	27.08±1.69	<0.001
R10	49.08±5.77	45.08±6.96	<0.001
R30	8.21±0.67	13.03±1.38	<0.001
R50	3.55±0.24	4.30±0.40	<0.001
R70	2.12±0.13	2.28±0.14	<0.001

BROAD-RT, Biaxially rotational dynamic radiation therapy; co-VMAT, Coplanar volumetric-modulated arc therapy; SD, Standard deviation; GTV, Gross tumor volume; CI, Confidence interval; CTV, Clinical target volume; PTV, Planning target volume; PRV, Planning organ at risk volume; PTV-GTV5, The volume after subtracting gross tumor volume plus 5 mm from the planning target volume of the prostate; D2cm, Maximum dose 2 cm away from PTV_all; R50, 50% isodose line volume divided by the planning target volume (prostate plus seminal vesicles); R70, 70% isodose line volume divided by the planning target volume (prostate plus seminal vesicles). DX%, dose to X% of the target or OAR volume; DXcc, dose to X cc of the target or OAR volume.

### Results of dosimetric quality assurance, total monitor units, and delivery times

The average (± standard deviation [SD]) passing rates were 98.2 ± 0.7% for BROAD-RT and 99.0 ± 1.0% for co-VMAT.

The average (± SD) prescribed MUs were 2963.1 ± 276.4 and 3494.7 ± 576.9 MU for BROAD-RT and co-VMAT, respectively (*p* < 0.001).

The average (± SD) delivery times were 619.5 ± 17.6 and 398.1 ± 64.3 s for BROAD-RT and co-VMAT, respectively (*p* < 0.001).

## Discussion

Here, we performed a planning study to investigate dosimetric benefits of non-coplanar VMAT using BROAD-RT over co-VMAT in prostate SBRT with focal boost to IPDLs. To our best knowledge, this is the first dosimetric planning study of prostate SBRT with focal boost using BROAD-RT. The prescribed dose to each target and dose constraints regarding OARs were the same as those used in the hypo-FLAME phase 2 study [21]. As a result of comparison, the use of non-coplanar VMAT using BROAD-RT significantly improved the dose distribution, such as normal tissue dose outside of PTVs (D2cm, R30, R50, and R70), high-dose expansion outside of GTV but within PTV (D0.03 cc of PTV-GTV5), and dose for femoral heads (D5% and mean dose), and significantly reduced total MUs. In addition, owing to the ability to deliver sequential non-coplanar beams without the need to rotate the couch, BROAD-RT enabled practical implementation of non-coplanar beams in VMAT.

It has been suggested that the application of non-coplanar beams could improve the dose distribution [27–29]. According to a dosimetric analysis of prostate SBRT using a C-arm linear accelerator without focal boost, adding supplementary non-coplanar beams to co-VMAT significantly reduced the dose to the rectum (*p* < 0.001) and the mean dose of the bladder (*p* < 0.001) compared with co-VMAT (without supplementary non-coplanar beams) [28]. Similarly, according to a dosimetric study by Miura et al., which compared non-coplanar with co-VMAT in conventional fractionated IMRT without focal boost, the use of non-coplanar VMAT significantly reduced the medium dose to OARs compared with co-VMAT, in which BROAD-RT using the Vero4DRT system (Mitsubishi Heavy Industries, Ltd., Japan) was applied as non-coplanar VMAT beam delivery [29]. Specifically, non-coplanar VMAT plans resulted in a significantly lower dose for the bladder with an average reduction of 2.6% in V10Gy (*p* = 0.0447), 6.8% in V20Gy (*p* < 0.001), 5.4% in V30Gy (*p* < 0.001), and 1.8% in V40Gy (*p* < 0.001), lower dose for the rectum wall with an average reduction of 1.6% in V40Gy (*p* = 0.0055), and lower mean dose for the bilateral femoral heads (*p* < 0.05).

In the current study, we performed non-coplanar VMAT via BROAD-RT with 2 arcs beams from the OXRAY system. The trajectory of the 2 arcs was arranged to form a “waving X shape,” with the intention of maximizing the non-coplanar component (Fig. 2). For planning, we aimed to minimize high-dose expansion outside of GTV but within PTV to avoid RT-related toxicities, evaluated as D0.03 cc of PTV-GTV5, while maintaining the dose coverage of GTV. Compared with co-VMAT, BROAD-RT　significantly reduced high-dose expansion outside of GTV but within PTV (*p* = 0.0067). Although there was no significant difference in high-dose application to the rectum or urethra, these results suggest that BROAD-RT has the potential to achieve safe dose escalation to IPDLs, which would be particularly important in cases where they are located close to the urethra or rectum. Regarding OARs, no significant differences were observed in high-dose exposure to OARs, and medium-dose exposure to the rectum and penile bulb was slightly increased in the BROAD-RT plan due to the use of a non-coplanar trajectory, as expected. However, the femoral head dose was successfully reduced (*p* < 0.001 for both D5% and mean dose), considered to be because the beam trajectory of BROAD-RT directly avoided the femoral heads. In addition, dose distributions outside of PTV were significantly improved in BROAD-RT. Specifically, D2cm (*p* < 0.001), R30 (*p* < 0.001), R50 (*p* < 0.001), and R70 (*p* < 0.001) were significantly improved. Further investigations are needed to evaluate the clinical impact of these dosimetric advantages in the setting of prostate SBRT with focal boost.

One of the main strengths of BROAD-RT is its ability to deliver sequential non-coplanar beams without the need to rotate the couch, made possible by simultaneously rotating the gantry and O-ring around two axes. Compared with the frequently used C-arm linear accelerator, which needs the rotation of the couch, BROAD-RT enables practical implementation of non-coplanar beams in VMAT. In this regards, BROAD-RT is considered as a promising non-coplanar VMAT method. Despite the ease of applying non-coplanar VMAT using BROAD-RT, the main trade-off was the extension of the beam delivery time. The current study showed a 3.7-minute extension of the delivery time in BROAD-RT; the average delivery times were 619.5 and 398.1 s for BROAD-RT and co-VMAT, respectively (*p* < 0.001). As the intra-fractional prostate position error increases along with the extensions of treatment time [37–39], prolongation of the treatment time may result in decreased treatment accuracy. Therefore, CBCT-based repositioning of the patient during treatment (e.g. before starting irradiation of the second arc) may be beneficial in minimizing the intra-fractional prostate position errors.

We acknowledge that the current study has several limitations. Firstly, we did not evaluate the dosimetric benefit of BROAD-RT regarding the location of IPDLs in the prostate gland, making it difficult to judge which location of IPDLs benefits most from this novel RT method of SBRT with focal boost. Secondly, as always accompanies planning studies regarding IMRT, the imbalance of efforts in the optimization process between the two arms would interfere with the results of comparison. In the current study, to minimize this bias, the optimization process of both IMRT plans was started using the same template, and it was repeated until the treatment plan met the dose constraints. Furthermore, although we used a “waving X shape” 2 arc as non-coplanar trajectory to maximize the non-coplanar component, we did not perform analysis to determine the best non-coplanar trajectory for prostate SBRT with focal boost. Therefore, further studies are needed to ascertain the most beneficial trajectory for prostate SBRT with focal boost. Due to those limitations, our findings regarding the merit of non-coplanar VMAT using BROAD-RT are not conclusive but merely hypothesis-generating. Nevertheless, considering the growing clinical demand for SBRT in prostate radiotherapy and expectations regarding focal boosting as a promising method for improving disease control, our findings may be particularly informative.

In conclusion, non-coplanar VMAT via BROAD-RT improved the dose distribution, mainly outside of the PTV and for some of the organs at risks, in prostate SBRT with focal boost compared with coplanar VMAT. As BROAD-RT enables practical implementation of non-coplanar VMAT, it is considered a promising RT method of prostate SBRT with focal boost. Further studies to determine the optimal non-coplanar trajectory are warranted.

## Supplementary Information

Supplementary Material 1: Prescriptions and dose constraints of targets and organs at riskused in the hypo-FLAME study [21]. GTV, gross tumor volume; CI, confidence interval; CTV, clinical target volume; PTV, planning target volume; PRV, planning organ at risk volume.

## Data Availability

The data that support the findings of this study are available from the corresponding author (TM) on reasonable request.

## References

[1] Bray F, Laversanne M, Sung H, Ferlay J, Siegel RL, Soerjomataram I et al. Global cancer statistics 2022: GLOBOCAN estimates of incidence and mortality worldwide for 36 cancers in 185 countries. CA Cancer J Clin. 2024. 10.3322/caac.21834 38572751

[2] Sasaki T, Higashi T, Inoue T. Urological cancer statistics on incidence from 1975 to 2019 and mortality from 1958 to 2022 in Japan. Int J Clin Oncol. 2024;29:1088–95. 38954076 10.1007/s10147-024-02575-3

[3] National Comprehensive Cancer network. NCCN guidelines; prostate cancer version 1. 2025. The category of prostate cancer. 2025. https://www.nccn.org/guidelines/category_1.

[4] Kohjimoto Y, Uemura H, Yoshida M, Hinotsu S, Takahashi S, Takeuchi T, et al. Japanese clinical practice guidelines for prostate cancer 2023. Int J Urol. 2024;31:1180–222. 39078210 10.1111/iju.15545

[5] Hatano K, Tohyama N, Kodama T, Okabe N, Sakai M, Konoeda K. Current status of intensity-modulated radiation therapy for prostate cancer: history, clinical results and future directions. Int J Urol. 2019;26:775–84. 31115116 10.1111/iju.14011

[6] Nakamura K, Nihei K, Saito Y, Shikama N, Noda SE, Hara R, et al. A Japanese multi-institutional phase II study of moderate hypofractionated intensity-modulated radiotherapy with image-guided technique for prostate cancer. Int J Clin Oncol. 2024;29:847–52. 38630382 10.1007/s10147-024-02517-z

[7] Aizawa R, Takayama K, Nakamura K, Inoue T, Yamasaki T, Kobayashi T, et al. Ten-year outcomes of high-dose intensity-modulated radiation therapy for nonmetastatic prostate cancer with unfavorable risk: early initiation of salvage therapy may replace long-term adjuvant androgen deprivation. Int J Clin Oncol. 2019;24:1247–55. 31152322 10.1007/s10147-019-01478-y PMC6736780

[8] Aizawa R, Takayama K, Nakamura K, Inoue T, Yamasaki T, Kobayashi T, et al. Low incidence of late recurrence in patients with intermediate-risk prostate cancer treated by intensity-modulated radiation therapy plus short-term androgen deprivation therapy. Int J Clin Oncol. 2020;25:713–9. 31820209 10.1007/s10147-019-01596-7

[9] Nakamura K, Ikeda I, Inokuchi H, Aizawa R, Ogata T, Akamatsu S et al. Long-term outcomes of a prospective study on highly hypofractionated intensity modulated radiation therapy for localized prostate cancer for 3 weeks. Pract Radiat Oncol. 2023. 10.1016/j.prro.2023.06.004 37414247

[10] Aizawa R, Inokuchi H, Ikeda I, Nakamura K, Ogata T, Akamatsu S, et al. Impact of prostate position-based image-guidance in intensity-modulated radiation therapy for localized prostate cancer. Int J Clin Oncol. 2024;29:325–32. 38191958 10.1007/s10147-023-02456-1

[11] Aizawa R, Otani T, Ogata T, Moribata Y, Kido A, Akamatsu S, et al. Spatial pattern of intraprostatic recurrence after definitive external-beam radiation therapy for prostate cancer: implications for focal boost to intraprostatic dominant lesion. Adv Radiat Oncol. 2024;9:101489. 38681892 10.1016/j.adro.2024.101489 PMC11043806

[12] Chopra S, Toi A, Taback N, Evans A, Haider MA, Milosevic M, et al. Pathological predictors for site of local recurrence after radiotherapy for prostate cancer. Int J Radiat Oncol Biol Phys. 2012;82:e441–8. 22284038 10.1016/j.ijrobp.2011.05.035

[13] Aizawa R, Mizowaki T. Narrative review of focal boost to intraprostatic dominant lesion in intensity-modulated radiation therapy for localized or locally advanced prostate cancer. Int J Clin Oncol. 2025;30:1448–62. 40483656 10.1007/s10147-025-02799-x PMC12297006

[14] Poon DMC, Yuan J, Yang B, Kerkmeijer LGW, Kishan AU, Murthy V, et al. Magnetic resonance imaging–guided focal boost to intraprostatic lesions using external beam radiotherapy for localized prostate cancer: a systematic review and meta-analysis. Eur Urol Oncol. 2023;6:116–27. 41429687 10.1016/j.euo.2022.10.001

[15] Kerkmeijer LGW, Groen VH, Pos FJ, Haustermans K, Monninkhof EM, Smeenk RJ, et al. Focal boost to the intraprostatic tumor in external beam radiotherapy for patients with localized prostate cancer: results from the FLAME randomized phase III trial. J Clin Oncol. 2021;39:787–96. 33471548 10.1200/JCO.20.02873

[16] Aizawa R, Ogata T, Goto T, Nakamura K, Takayama K, Ashida R, et al. Highly hypofractionated biaxially rotational dynamic radiation therapy (BROAD-RT) for high-risk prostate cancer. Cancer Sci. 2025;116:1004–11. 39834115 10.1111/cas.16429 PMC11967259

[17] Ashida R, Nakamura K, Aizawa R, Iramina H, Takayama K, Nakamura M, et al. Highly hypofractionated intensity-modulated radiation therapy for nonmetastatic prostate cancer with a simultaneous integrated boost to intraprostatic lesions: a planning study. Jpn J Radiol. 2022;40:210–8. 34350542 10.1007/s11604-021-01186-6

[18] Kim YJ, Yoon KJ, Kim YS. Simultaneous integrated boost with stereotactic radiotherapy for dominant intraprostatic lesion of localized prostate cancer: a dosimetric planning study. Sci Rep. 2020;10:14713. 32895442 10.1038/s41598-020-71715-2 PMC7477222

[19] Miralbell R, Roberts SA, Zubizarreta E, Hendry JH. Dose-fractionation sensitivity of prostate cancer deduced from radiotherapy outcomes of 5,969 patients in seven international institutional datasets: alpha/beta = 1.4 (0.9–2.2) Gy. Int J Radiat Oncol Biol Phys. 2012;82:e17–24. 21324610 10.1016/j.ijrobp.2010.10.075

[20] Morgan SC, Hoffman K, Loblaw DA, Buyyounouski MK, Patton C, Barocas D, et al. Hypofractionated radiation therapy for localized prostate cancer: an ASTRO, ASCO, and AUA evidence-based guideline. J Clin Oncol. 2018;36:3411–34. 10.1200/JCO.18.01097 PMC6269129 30307776

[21] Draulans C, Haustermans K, Pos FJ, van der Heide UA, De Cock L, van der Voort J, et al. Stereotactic body radiotherapy with a focal boost to the intraprostatic tumor for intermediate and high risk prostate cancer: 5-year efficacy and toxicity in the hypo-FLAME trial. Radiother Oncol. 2024;201:110568. 39362607 10.1016/j.radonc.2024.110568

[22] Smyth G, Evans PM, Bamber JC, Mandeville HC, Welsh LC, Saran FH, et al. Non-coplanar trajectories to improve organ at risk sparing in volumetric modulated arc therapy for primary brain tumors. Radiother Oncol. 2016;121:124–31. 27481571 10.1016/j.radonc.2016.07.014

[23] Ma M, Ren W, Li M, Niu C, Dai J. Dosimetric comparison of coplanar and noncoplanar beam arrangements for radiotherapy of patients with lung cancer: a meta-analysis. J Appl Clin Med Phys. 2021;22:34–43. 33634946 10.1002/acm2.13197 PMC8035566

[24] Woods K, Nguyen D, Tran A, Yu VY, Cao M, Niu T, et al. Viability of non-coplanar VMAT for liver SBRT as compared to coplanar VMAT and beam orientation optimized 4pi IMRT. Adv Radiat Oncol. 2016;1:67–75. 27104216 10.1016/j.adro.2015.12.004 PMC4834900

[25] Ono Y, Yoshimura M, Hirata K, Ono T, Hirashima H, Mukumoto N, et al. Dosimetric advantages afforded by a new irradiation technique, dynamic WaveArc, used for accelerated partial breast irradiation. Phys Med. 2018;48:103–10. 29728221 10.1016/j.ejmp.2018.03.015

[26] Uto M, Mizowaki T, Ogura K, Miyabe Y, Nakamura M, Mukumoto N, et al. Volumetric modulated dynamic WaveArc therapy reduces the dose to the hippocampus in patients with pituitary adenomas and craniopharyngiomas. Pract Radiat Oncol. 2017;7:382–7. 28666908 10.1016/j.prro.2017.04.004

[27] Price RA, Hanks GE, McNeeley SW, Horwitz EM, Pinover WH. Advantages of using noncoplanar versus axial beam arrangements when treating prostate cancer with intensity-modulated radiation therapy and the step-and-shoot delivery method. Int J Radiat Oncol Biol Phys. 2002;53:236–43. 12007964 10.1016/s0360-3016(02)02736-0

[28] Sharfo AWM, Rossi L, Dirkx MLP, Breedveld S, Aluwini S, Heijmen BJM. Complementing prostate SBRT VMAT with a two-beam non-coplanar IMRT class solution to enhance rectum and bladder sparing with minimum increase in treatment time. Front Oncol. 2021;11:620978. 33816253 10.3389/fonc.2021.620978 PMC8018286

[29] Miura H, Nakao M, Doi Y, Ozawa S, Kenjo M, Nagata Y. Treatment planning comparison between dynamic wave arc and volumetric modulated arc therapies for prostate-cancer treatment. Med Dosim. 2022;47:48–53. 34538693 10.1016/j.meddos.2021.08.001

[30] Hirashima H, Adachi H, Ono T, Nakamura M, Ono Y, Iwai T, et al. Determination of patient-specific trajectory for biaxially rotational dynamic-radiation therapy using a new O-ring-shaped image guided radiotherapy system. Phys Imaging Radiat Oncol. 2025;33:100698. 40123770 10.1016/j.phro.2025.100698 PMC11926428

[31] Hiraoka S, Hirashima H, Nakamura M, Tanaka F, Adachi H, Ono Y, et al. Integration test of biaxially rotational dynamic-radiation therapy for nasopharyngeal carcinoma: efficacy evaluation and dosimetric analysis. Radiother Oncol. 2025;207:110879. 40189151 10.1016/j.radonc.2025.110879

[32] Hirotaki K, Tomizawa K, Kitou S, Jinno S, Moriya S, Fujisawa T et al. Dosimetric comparison of non-coplanar VMAT without rotating the patient couch versus conventional coplanar/non-coplanar VMAT for head and neck cancer: First report of Dynamic Swing Arc. Adv Radiation Oncol. 2024. 10.1016/j.adro.2024.101706 PMC11794065 39911721

[33] Hirotaki K, Motegi K, Moriya S, Wakabayashi M, Sakae T, Ito M, et al. Dosimetric and robustness evaluation of biaxially rotational dynamic radiation therapy with robust optimization. J Appl Clin Med Phys. 2025;26:e70194. 40778744 10.1002/acm2.70194 PMC12332889

[34] Weinreb JC, Barentsz JO, Choyke PL, Cornud F, Haider MA, Macura KJ, et al. Eur Urol. 2016;69:16–40. PI-RADS prostate imaging - reporting and data system: 2015, Version 2. 10.1016/j.eururo.2015.08.052 PMC6467207 26427566

[35] Kanda Y. Investigation of the freely available easy-to-use software ‘EZR’ for medical statistics. Bone Marrow Transpl. 2013;48:452–8. 10.1038/bmt.2012.244 PMC3590441 23208313

[36] Miften M, Olch A, Mihailidis D, Moran J, Pawlicki T, Molineu A, et al. Tolerance limits and methodologies for IMRT measurement-based verification QA: recommendations of AAPM task group 218. Med Phys. 2018;45:e53–83. 29443390 10.1002/mp.12810

[37] Langen KM, Willoughby TR, Meeks SL, Santhanam A, Cunningham A, Levine L, et al. Observations on real-time prostate gland motion using electromagnetic tracking. Int J Radiat Oncol Biol Phys. 2008;71:1084–90. 18280057 10.1016/j.ijrobp.2007.11.054

[38] Ikeda I, Mizowaki T, Ono T, Yamada M, Nakamura M, Monzen H, et al. Effect of intrafractional prostate motion on simultaneous boost intensity-modulated radiotherapy to the prostate: a simulation study based on intrafractional motion in the prone position. Med Dosim. 2015;40:325–32. 26002121 10.1016/j.meddos.2015.04.003

[39] Higuchi D, Ono T, Kakino R, Aizawa R, Nakayasu N, Ito H, et al. Evaluation of internal margins for prostate for step and shoot intensity-modulated radiation therapy and volumetric modulated arc therapy using different margin formulas. J Appl Clin Med Phys. 2022;23:e13707. 35719051 10.1002/acm2.13707 PMC9512338

